# Bibliographical Notices

**Published:** 1846-08

**Authors:** 


### BIBLIOGRAPHICAL NOTICES.

*A Practical Treatise on the Diseases of Children.* By JAMES MILMAN COLEY, M. D., Member of the Royal College of Physicians, &c. Author of a Treatise on the Remittent Fever of Infants, &c., and of Essays on Phlegmonous Erysipelas, on Scherroide, Keloid et Cancroid, &c. 8vo. pp. 467. London, 1846.

As long almost as we can recollect anything of professional moment, we have been familiar with the "Practical Treatise on the remittent fever of infants, with remarks on hydrocephalus, &c., by James M. Coley," which was published "in the provinces," at Stamport, in 1813,—thirty-three years, that is, ago. Dr. Coley is, therefore, a veteran in the practice, if not in the science of his profession. We are told, indeed, in the Introduction, that the practical portions have been the result of an experience of forty years, during which time the author "has been constantly engaged in recording cases, accumulating facts, and pursuing pathological inquiries."

His "first love"—the essay on the remittent fever of infants—probably suggested to him the present "treatise," stimulated perhaps by the fact of his having become a metropolitan practitioner, and the consequent necessity—for it is so esteemed by many—of heralding the advent of a new comer by some publication that may serve as an advertisement to the citizens of London.

Even in the introduction to the work, we meet with assertions which make us doubt whether the author possesses any marked fitness for elucidating the comprehensive subject which he has selected as his "prolegomenon"—if the work be really intended to usher him into metropolitan notice. The diseases of children—he admits, as he necessarily must do—have attracted the attention of physicians in all countries, "many of whom have published useful essays on the subjects." Amongst these he enumerates but one American—"*Dewes.*" Of the writings of Eberle, or the still later of Drs. Stewart and Condie,—the last of whom has published an excellent and plenary work on the subject—and we mean, by this remark, no disparagement of the others—seem to be totally unknown to him. We are not surprised, therefore, at the admission,—"I am not aware that any author—British or Foreign—has published a work comprehending all diseases incident to children, and their appropriate surgical as well as medical treatment. This omission may be accounted for by the division of the profession, which has limited the education and practice of physicians, who have hitherto been the principal and only writers on infantile disorders." p. xiii. This division does not, however, apply to the profession in the United States.

"The attention of pathologists," he remarks in his preface, "has of late been much engaged by the important subject of Tuberculization; some contending that it is the result of inflammation, and others of an opposite condition. The disposition to this morbid process, as I have stated, may be hereditary or acquired; but I have endeavoured to show, that its development is dependant on vascular excitement, produced by external injury or atmospherical vicissitude, and that the process by which the morbid product is deposited is of an inflammatory nature. The prophylactic cure and antiphlogistic treatment, which this theory suggests, will often be found to possess the advantage of modifying or averting the specific production, especially in that form of the disease, which we denominate scrophula." p. xvi.

Yet nothing has been more firmly established, in our opinion, than that the morbid action in scrophulosis and tuberculosis differs very materially from the inflammatory process. The facts which hæmatology have brought forth; the different phenomena presented by those affections, and the most successful hygienic and therapeutical agencies in each have sufficiently shown, that both tuberculosis and scrophulosis consist in perverted nutrition, but certainly not in that particular form, which characterizes inflammation. Somewhat analogous, however, they must be in their local manifestations, if our view be correct, that inflammation is a disease of the system of nutrition of the part affected. Our experience has satisfied us, that in place of an antiphlogistic treatment being appropriate for those who are predisposed to tuberculosis or scrophulosis, an opposite course should be adopted; the patient should be placed on a regimen, which will develope rather than diminish his powers; and even if experience had not shown this to be the correct view, we should have been induced to embrace it from the facts elicited by the observation of recent hæmatologists.

On one more observation in the preface we may briefly animadvert. "The few philological remarks," says the author, "which occur in the course of the work, have been introduced for the purpose of correcting an erroneous etymology, which has in some instances been copied by successive compilers, and led to improper views and practice." p. xvi. Yet we think Dr. Coley has not been eminently successful in this department; nor has he bestowed much befitting knowledge or attention in this volume on what may be regarded as one branch of philolology,—the correct mode of writing classical terms and prescriptions. Thus we have "*Hydrocephalus externus* or *Cephalæmatoma*,"—no one—we had presumed—regarding these terms as synonymes. *Potassæ Iodidi* for *Potassii Iodidi; Tinia Tarsi* for *Tinea Tarsi*—an obsolete term, by the way; *Acidi Hydrochloridi* for *Acidi Hydrochlorici; Adepis* passim for *Adipis;* Εκζημα for Εκζεμα; *Nœvus* for *Nævus;* "*Muguet* or *Mucosity*;" *Phagadena* for *Phagedæna; Dyptherite* for *Diphtherite;* "*Acute muco-enteritis* or *Diarrhœa;*" *Phthysis* for *Phthisis; Albuminaria* for *Albuminuria; Phymosis* for *Phimosis;* &c. &c. These errors—some of which are doubtless typographical—would not have been signalized by us, had not the author taken credit for his accuracy. The proper names are sadly mutilated,—some past all recognition. Dr. Bacton means, we presume, Dr. Barton; and Dr. Hildreth of *Tancaville* is, doubtless, Dr. Hildreth of Zanesville, the gentleman whose name has already undergone such unaccountable metamorphoses; and who appears to be best known in the European Journals as Dr. *Tanesville!*

We do not think the work of Dr. Coley an important addition to our medical libraries on this side of the Atlantic. We are, indeed, liberally supplied with publications on the subject by our own authors; and we think the literature of Great Britain would have been more enriched by the republication of one of these—that of Dr. Condie—than by the one before us. Dr. Coley has evidently consulted—and with due acknowledgments—the productions of some of the modern French writers, and especially the excellent treatise of MM. Rilliet and Barthez; but he has not been—as the best writers on this side of the Atlantic are—cosmopolitan in his researches; and consequently his work is less complete than it might have been. In his direction for treatment he errs greatly in not laying down his indications; hence, we must suppose that particular drugs are adapted for the removal of particular pathological conditions. Take for example chorea.

"*Treatment.* The variety arising from constipation should be attacked by the exhibition of castor oil or chloride of mercury and jalap, which should be repeated every second or third morning"—as if not cathartics in general but these special cathartics were indicated. Again. "The neuralgic and periodical symptoms will be removed by five minims of liquor potassæ arsenitis, given in some convenient vehicle, three times a day." "The anæmii [anæmic] and chlorotic varieties will be most successfully combated by two grains of sulphate, or twenty of oxyde, of iron, repeated three times every day." His pathology may be imagined but not *comprehended* by the statement, that these medicines operate curatively "by restoring the hæmatosine and fibrine [?] of the blood, and invigorating the abdominal ganglionic nerves."!

The well informed pathologist and therapeutist will certainly not be disposed to take Dr. Coley as a *facilè princeps.* His mode, indeed, of viewing disease in most of its aspects is scarcely *à portée* with the existing condition of medical science; and with this observation we take leave of him. To our publishing undertakers—*entrepreneurs*—who are so anxious to reproduce everything that appears abroad, we would say, for their own sakes, "*requiescat in pace*"*!*

SYDENHAM SOCIETY. *An Anatomical Description of the Diseases of the Organs of Circulation and Respiration.* BY CHARLES EWALD HASSE, M. D., Professor of Pathology and Clinical Medicine at the University of Zurich, &c. &c. *Translated and edited by* W. G. SWAINE, M. D., Physician Extraordinary to H. R. H. the Duchess of Kent. 8vo. pp. 400. London, 1846.

On all subjects that demand elaborate investigation, the Germans hold certainly an elevated position in the scale of nations. Their commentaries on the classics of Greece and Rome; their admirable philological enquiries into the languages of those ancient countries, and, indeed, of all countries—ancient and modern—in every quarter of the globe; their labours in astronomical science; in mathematics, simple and applied,—their intimate investigations in chemistry in all its branches, but especially in organic chemistry and physiology, have acquired them a world-renown; and when we observe—emanating from one of them—a treatise like that before us, on a subject requiring pains-taking and accurate observation, we hail its appearance, confident, that a large amount of valuable matter must be brought together: nor—in the present instance—are we likely to be mistaken.

Dr. Hasse has long devoted himself to the study of pathological anatomy, and is possessed of a mind capable of profiting by such application.

"My former fellow student," says the Editor, "and I have both pride and pleasure in adding, my intimate friend, Professor E. Hasse, conceived a very early predilection for pathological anatomy, and this bias was fostered and matured by a lengthened sojourn at the schools and hospitals of Paris and Vienna. On his return to his own university, Leipsic, he was appointed by my revered clinical instructor, Professor Clarus, to be assistant clinical teacher, and also pathological prosector at the principal hospital. With such means at his disposal, my friend forthwith commenced forming a pathological connection, which, under his auspices, has grown into a most interesting and valuable museum; and the present work is but a collateral result of the unwearied *practical industry* which he displayed in that undertaking, combined with a thorough knowledge of all that other observers had before achieved in the same field of science.

Hence it will at once be seen that this treatise differs essentially from what is commonly called a compilation. The high estimation in which it is held in Germany is clearly shown by the fact, that since its publication, Professor Hasse has had the offer of the chair of clinical medicine from no fewer than five universities. He has accepted that vacated by Professor Schönlein at Zurich, and at present holds the additional rank of Rector of that Germano-Swiss University." p. vi.

It was on account of the high reputation of Professor Hasse, and the acknowledged excellence of the volume before us, that the council of the Sydenham Society of London determined to have the original translated, and issued under their auspices,—as one of the valuable works with which they are favouring the profession. The work has been revised by the author, who has re-written some of the most important chapters, and added much information concerning the microscopic characters of a great variety of diseases.

The volume—as the title states—contains only the pathological anatomy of the organs of circulation and respiration:—

"The original is intended by the author as the first of a series of tomes, comprising the diseases of every system and organ of the body. But the uncertainty that necessarily attaches to the appearance of comprehensive works in distinct parts, has induced the council to prefer publishing the present volume, which constitutes singly a complete and valuable treatise, as a separate and independent work." p. vii.

Part First—"*Diseases of the Organs of Circulation*"—consists of five chapters, embracing respectively—Diseases of the lymphatic vessels and glands; Diseases of the veins; Diseases of the arteries: Heterologous formations and diseases of the heart. Part Second—Diseases of the organs of respiration—has fifteen chapters—treating, respectively, of disease of the pleural membrane; inflammatory disease of the substance of the lung; diseases of the lung not necessarily dependent upon, or allied to, inflammation—as gangrene, œdema, &c.; disease of the air-passages; inflammatory disease of the air-passages; exudative inflammation of the air-passages; catarrhal pneumonia; organic sequelæ of catarrh; emphysema of the lung; tubercular disease; cancerous tumours in the respiratory organs; formation of cysts in the respiratory organs; pseudomelanosis of the lungs and bronchial glands; diseases of the thymus gland; and diseases of the thyroid—a division, by the way, not eminently marked by *lucidus ordo.*

Our space and objects will not permit more than a notice of this volume, which must be esteemed a valuable addition to the library of the pathologist; but it would have been still more valuable had its author attended more to the labours of observers who write in the English branch of the great Teutonic stem of languages. We would instance the chapter on diseases of the thymus gland as wanting in much exact information, which might have been readily culled from Sir A. Cooper, Mr. Simon, and from observers on this side of the Atlantic.

The work is beautifully got up;—in the same elegant manner, indeed, as its predecessors from the press of the Sydenham Society of London;—of which it may be said, "*florescet quoti die magis.*"

---

*Scrofula; Its Nature, its Causes, its Prevalence, and the Principles of Treatment.* By Benjamin Phillips, F. R. S., Assistant Surgeon to the Westminster Hospital. 8vo. pp. 350. Lea & Blanchard, Philadelphia, 1846.

The work before us was published in London within the present year, and is now reprinted for the use of the profession in this country, by our enterprising townsmen, to whom we are indebted for a large proportion of the standard works on Medicine and its collateral branches which have appeared in the English language within the last few years.

In the investigation of his subject, the author has manifestly spent much time, read extensively, and brought together many facts and opinions derived from a great variety of sources, and has thus given us a learned, although certainly not a very original work.

After speaking of the opinions entertained of the disease by those who have preceded him, the author proceeds, in chapter second, to state his "own ideas of the nature of Scrofula." "I conceive, then," he remarks, "that Scrofula is a disease of the Constitution, and that it is most clearly manifested by certain external signs, of which swelling of the subcutaneous lymphatic ganglia is the most conclusive. But *tumid glands*, however, wherever they may be situated, are not always a proof that a constitution is scrofulous; they may be the result of local irritation, in an apparently sound constitution. The glands in the groin may swell, from a sore on the feet; a mesenteric gland may swell under the influence of an ulcer in the intestine; a cervical gland may enlarge under the irritation of teething, or of scalp disease. A *tumid gland*, even *in the neck*, is then no proof that the constitution of the individual in whom it is found is scrofulous. But supposing one, or several cervical glands to become tumid, in the apparent absence of any obvious local irritation, this would constitute a strong ground for suspicion, that the constitution was suffering under the taint of Scrofula. It would not, however, amount to more than suspicion, and the suspicion could scarcely receive absolute confirmation, unless we have the opportunity of observing the contents of the tumor itself. Unless the swelling of the gland be accompanied by the deposite of a product, hereafter to be described, known as *scrofulous matter*, the proof of a scrofulous constitution is in my judgment, wanting."

Mr. Phillips seems to place little confidence in what are called temperaments, in predisposing to the disease. "The result of my own observation of persons whose constitutions are tainted with Scrofula, has satisfied me that there is the utmost possible variety in the external characters of those who present undoubted scrofulous taint. When the taint is made evident by scrofulous deposits, we find in one case the hair and complexion are dark, in another light; in one the cheeks are rosy, in another pale; in one, the alæ nasi are expanded and the upper lip is tumid; in another, both of those features present opposite characters. So that it becomes a matter of great difficulty to determine whether the presence or absence of those signs, is most characteristic of a scrofulous taint."

He believes "that diseases regarded as scrofulous, but in which no scrofulous matter is present, are not scrofulous at all, but simply the result of such low inflammatory action as is often set up in a debilitated state of the constitution," and that he knows of "no certain sign by which the state of the constitution which precedes the deposit of scrofulous matter can be recognized."

"In a constitution favourable for the deposite of scrofulous matter, I believe there are no features, in the absence of the tumor, so constant and so conclusive as to justify a reliance upon them, in pronouncing an opinion whether a constitution be scrofulous or not."

After denying so emphatically that the presence of any or all of the symptoms usually enumerated as characteristic of Scrofula, and insisting that nothing but the presence of the "scrofulous deposite" can be regarded as conclusive evidence of the existence of the disease, it surely was incumbent on an author, writing expressly on the subject, to have endeavoured to determine the nature, composition and appearance of the deposite, so that no difficulty could arise in discriminating between it and other products of disease, and yet, Mr. Phillips has signally failed to do so, and, indeed, seems to have taken but little pains on the subject. Contenting himself with the observations of others, without even verifying them himself, we have the descriptions of Albers, Ruette, Bredow, Dalrymple, and Gulliver, which are as unlike as the names of the authors. It is not only in regard to the physical properties, as observed by the aid of the microscope, that the subject is thus lightly passed over, but the "Chemical characters" of the deposite are disposed of in the same summary way. A single page contains all that is said on this point, and that consists wholly of the heterogenous observations of other inquirers; for example: "Prout regards this matter as albumen, incompletely developed. Gendrin as a mass of albumen, with excess of salts. L'Héritier found it to contain albumen, very soft fibrin, some fatty matter, and carbonate and phosphate of lime. Bredow regards the matter as albuminate of potash, or soda."

Can any thing be more unsatisfactory? And this in a book written professedly on the subject, in which the deposite is regarded as so specific as to characterise the disease, independently of all other symptoms!

The author admits that the state of the organ in which the deposite is about to take place undergoes some change or preparation. "Commonly, if not always, glandular structures do undergo considerable change before scrofulous matter is deposited in them. They acquire a considerable increase in volume, in density, and in vascularity." But whether this state of a gland is determined by the circulation within it of blood which has undergone a change, or is independent of the blood—whether the blood fits the organ to receive the deposite, or the organ fits itself, is a question which he regards as of very difficult solution. The greater frequency with which particular glands are affected, he seems to regard as evidence that it cannot arise from the action of the blood, but must proceed from some other developing cause, such as irritation of the part, from the action of cold, bad chyle supplied by improper food, etc.

These doubts, it appears to us, are quite inconsistent with the doctrine advocated in other parts of the work, that the disease is constitutional, and dependent on a vitiated condition of the blood. Of this vitiated or changed condition of the blood, which he thinks precedes the deposite and gives occasion to it, he attempts no explanation, nor as to its chemical or structural change does he afford us the least new light. The sum of his conclusions on this point may be seen in the following closing remarks of the fifth chapter: "So much may, I think, be fairly assumed, that the blood is changed before the deposite is made, that the accumulation of certain morbid materials in the blood constitutes what is known as the scrofulous diathesis or constitution, and that their deposition in the subcutaneous lymphatic glands constitutes what we know as Scrofula."

The sixth chapter is occupied with a discussion of the question whether *pulmonary turbercle, or phthisis, and Scrofula,* in their nature are identical, and he takes the negative side, and this in disregard of the acknowledged fact that the deposite in both cases, whether seen by the naked eye, through a microscope, or tested by chemical analysis, presents no difference in its character and composition—whether taken from the mesenteric, cervical or inguinal glands, or the lungs, no difference can be discovered. The grounds on which he mainly relies in support of his proposition are "that in districts where the causes of phthisis act with most intensity, those of scrofula fall lightest; that the age when the ravages of scrofula are most keenly felt, is precisely that when the visitation of phthisis is least to be apprehended; that the sex which suffers most severely from one of these diseases is least affected by the other. And beyond all this, there is the fact that among the numerous victims of phthisis, at least eighteen out of every twenty exhibit no marks of having suffered from Scrofula."

Without meaning to affirm either side of the question, we may just remark that not one of the allegations of the author can be admitted to be at all conclusive. That Scrofula occurs least frequently in districts where phthisis most prevails, is a fact to be doubted; and if admitted, only proves that local causes operate to determine the developement of disease in one organ more than another, as in hot climates inflammation is caused in the abdominal viscera, in colder latitudes, in the thoracic, etc.

That a difference exists in the predisposition of different organs to be affected according to age and sex, is a fact of too general application to be adduced as evidence of a difference of character because of a difference in seat; and whether the disease occur in the lungs or the lymphatic glands, there, of course, on post mortem examination, we may expect to find the lesions of structure consequent upon it.

In reference to the *causes of Scrofula*, the author has collected, partly from his own researches, much valuable statistical information, which he has accompanied by some very judicious remarks. He admits that hereditary predisposition often exists, but seems to attach less importance to it than John Hunter, Lugol and others. Intermarriage, so much dwelt on by some, he discards altogether as a cause.—"My impression is, that intermarriages among healthy persons tend to no such calamity as the production of Scrofula; but I must not be understood to assert that other physical or mental influences may not result from such unions." Unfavourable hygienic influences, and especially a deficiency of nutritious food, are properly regarded as the most potent and common of the causes of Scrofula.

Mr. Phillips, besides the opportunities presented to him as a distinguished practising Surgeon in the great British metropolis, is attached to a large hospital in which the occasions for the exercise of his skill in the treatment of scrofulous diseases must have been numerous; his experience, therefore, with the use of the various curative agents commonly employed, is valuable,—although far less so than if recommended by a consideration of the pathology of the cases or the ascertained therapeutical action of the remedies themselves.

The articles and means most prominent in his estimation, and to which he has chiefly confined his remarks, are the following:

*Mercury.*—"I will not occupy time by considering the theories which have been invented to explain the action of Mercury, as the best of the remedies employed for the cure of Scrofula; but I will simply deny that it is an agent upon which we can rely for the cure of Scrofula. In the sense of a remedial agent, capable alone, and under ordinary circumstances, of removing Scrofula from the constitution, Mercury is not, I believe, entitled to any confidence; but in the sense of an agent to be variously associated with other medicines, according to the symptoms of the disease, there is no doubt but that it will be found useful in many cases of Scrofula. In some instances, in virtue of a purgative, in others of a general alterative influence. But I am satisfied that when so administered as to lower the general powers, whether by profuse purgation, or by salivation, its influence is usually, if not always, injurious."

*Iodine.*—"In my own practice I have exhibited every form of Iodine extensively in cases of Scrofula, and supposing the patient to remain exposed to the influence of the same conditions in which the disease was at first manifested, and the period of the year to be that which has not been found favourable for the cure of the disease under other modes of treatment, I cannot say that I have had reason to estimate the curative powers of Iodine so highly as many others have done. I know that among the out-patients of hospitals, whose circumstances remain unchanged, and who apply at the latter end of autumn, or the beginning of winter, we may often exhibit Iodine in every form for weeks or months, without producing any sensible amelioration in the patient's condition. I know also that at the beginning of summer, a patient similiarly affected and similarly treated, will, often in a few weeks, exhibit a marked improvement—but how much of this should be referred to Iodine? How much to season?

"I by no means wish to express the opinion that Iodine has no curative influence in Scrofula; and although I believe that it is not, ordinarily, strong enough to make head against the disease in an unfavourable season of the year, yet I think I have known some cases in which decided benefit has seemed to result from its use, even when the season and other circumstances have not been favourable, and when no change in those circumstances has occurred, beyond the exhibition of Iodine; and yet, even then, I refer the good to a general alterative action upon the economy, and not to any specific action; the general health has improved under the employment of the medicine, and the local disease has abated. Such cases have, however, formed a small minority of those in which Iodine has been administered by me, and I have endeavoured, though not satisfactorily, to account for these exceptional cases, by some change, some effort made by the system itself."

*Barium.*—"I do not mean to say that my experience of its power over Scrufola is such as to bear out the opinions of its efficacy so confidently expressed by Dr. Adair Crawford. But sure I am, that its power as a discutient, over scofulous glandular tumors, and over the scrofulous constitution, are little, if at all, inferior to those of Iodine. Its field of usefulness is, however, more limited than that of Iodine; because we have the advantage of a choice of many different combinations of that medicine. Barium yields only one preparation which has been much employed as a medicine; the meconnate and nitrate are very rarely used. Barium, however, seems to be a more certain stimulant than Iodine, or rather, we might say, irritant; and, in my judgment, its use is clearly contra-indicated where there is much free inflammatory excitability of the system; but in those cases where the tallow-like complexion, the pale tongue, and the languid circulation, accompanied by irritability of the mucous surfaces, are present, the virtues of the Barium are often very remarkably demonstrated. I usually give it in solution, a grain to an ounce of distilled water, with ten drops of Compound Tincture of Gentian. Of this solution, I commence with half an ounce twice a day, and on no occasion have I exceeded three grains in the day, and up to this moment I have not experienced any check in the administration of the medicine."

*Hydrochlorate of Lime.*—"Hufeland conceded to the Muriate of Lime similar properties, in relation to Scrofula, with those possessed by the Chloride of Barium, 'Except that it was more irritating, and therefore required to be used with more precaution.' I am not satisfied that it has any very evident action upon scrofulous glands. I cannot say that I have ever seen a case in which, in the absence of other influences, the discutient power of this medicine has been clearly manifested. But I am convinced that when given in moderate doses, it is more generally tolerated than the Chloride of Barium, and I therefore conclude that the inconveniences to which Hufeland was exposed, resulted from the mode in which he administered this medicine."

*Alkalies.*—To this class he attaches no great confidence.

"No one has made a larger trial of the Alkali than Brandish, and I think his statements with reference to it bear upon their face the marks of truth; and what is the result? That Caustic Potash, in large doses, continued for many months of several years and associated with good food, good air, and proper exercise, has seemed to cure many bad cases of Scrofula. Would they not have got well, and probably assoon without the Alkali? I believe in many instances they would.

"I have tried the medicine extensively, but not in such large doses as Brandish used, and I found the bitter ale a very convenient vehicle for its administration, and my experience is very similar to his. My conclusions drawn from that experience are, however, unlike those of Brandish. I have known many cases in which under this treatment the glandular tumors seemed to subside rather sooner than they would probably have done without it; but I have known many more in which it did not exercise any sensible effect."

*Burnt Sponge.*—"It is not necessary to inquire to which of the two substances, (Iodine and Animal Charcoal) its pretended virtues are owing, because we have never discovered that it really had any virtues; and in all the cases, and there are many, in which I had an opportunity of observing the effects of the medicine, I have never seen anything to satisfy me that it possesses any power over scrofulous swellings, or the scrofulous constitution."

*Cod-Liver Oil.*—"In the course of the last six years, I have employed it pretty extensively myself, and my estimate of its virtues, as a remedial agent in scrofula, is much less favourable than that of many others who have given it a trial. There is, however, scarcely any form of scrofula which I have not seen to improve under it; enlarged glands, sinuses, ulcers, lupus-like scrofula of the face, Caries, all these I have known to get better under its employment; but generally, one of two things happened, either the stomach or the patience failed before the remedy had been carried far enough to produce any considerable amelioration. Indeed, either my own patience or that of my patients, has usually given way long before they have consumed 100 lbs. or even 36 lbs. of the remedy; or have continued to take it for six months, or as many years, as some patients have done."

*Sea Water.*—"I have had opportunities of observing the internal employment of Sea-water, but not on a large scale, nor unassociated with residence on the coast. I have known it to be taken to the extent of a pint before breakfast, and to be attended with discomfort to the patient, from the profuseness of its purgative action; and when its daily use has been persisted in, the depressing effects which have resulted, have been injurious. But used daily, to the extent of a small tumbler, with an equal quantity of milk, and taken at bed-time, the patient submitted to this treatment has improved in health, in so far as the condition of the intestinal secretions can be taken to be a proof of the fact; but I am by no means satisfied that the time and the place have not had quite as decided an influence upon the patient's condition as the Sea-water introduced into the stomach.

"In order to drink Sea-water, persons have usually been taken to the sea-coast, and this at a favourable season of the year, and to those two circumstances, any good which has resulted from the practice may be attributed, I think, with more fairness, than to the daily ingestion of half a pint, or a pint of salt water."

*Mineral Waters.*—Our author does not deny that great benefit has accrued to Scrofulous persons from the employment of various mineral waters, but he justly infers that much of the credit of such cases was properly due to the change of season, change of air, diet, and other hygienic influences which were concurrently enjoyed.

"That they have been more indebted for the credit they possess to the enthusiasm of friends than to the faithful register of the cures, which it is alleged have resulted from their employment, is I think true. And no doubt M. Patissier was near the truth when he said, 'Les eaux minerales naturalles guerissent quelquefois, soulagent et consolent toujours.'"

